# Electrodeposition of nanocrystalline Fe_X_Co_1-X_ thin films from choline chloride–urea deep eutectic solvents

**DOI:** 10.3389/fchem.2025.1635084

**Published:** 2025-09-01

**Authors:** Tingjun Wu, Jiwon Kim, Yong-Ho Choa, Nosang V. Myung

**Affiliations:** 1 Xiamen Institute of Rare Earth Materials, Xiamen, Fujian, China; 2 Center for Advanced Materials, Institute for Advanced Engineering, Yongin, Gyeonggi, Republic of Korea; 3 Department of Materials Science and Chemical Engineering, Hanyang University, Ansan-si, Republic of Korea; 4 Department of Chemical and Biomolecular Engineering, University of Notre Dame, Notre Dame, IN, United States; 5 Department of Chemistry and Biochemistry, University of Notre Dame, Notre Dame, IN, United States

**Keywords:** deep eutectic solvent, choline chloride, urea, electrodeposition, CoFe, soft magnetic materials

## Abstract

Fe_x_Co_1-x_ thin films were electrodeposited from a choline–urea deep eutectic solvent (DES) containing Fe^3+^ and Co^2+^ ions under ambient conditions. Anomalous co-deposition was observed, with Fe preferentially depositing over Co. With higher cathodic potential, the film’s morphology shifted from smooth to nodular. X-ray diffraction (XRD) analysis showed iron oxide impurities at lower overpotential and temperatures (e.g., <−0.9 V at 70 °C), while impurity-free, nanocrystalline Co_50_Fe_50_ films had formed at higher temperatures (e.g., 130 °C). The films exhibited a body-centered cubic (BCC) structure with (110) preferred orientation and grain sizes of 30 nm–40 nm.

## Introduction

Soft magnetic materials, which can easily be magnetized or demagnetized, have many applications from magnetic storage media to recording head (Ikeda et al., 2010; Okada et al., 2002; Okada et al., 2004; Parkin et al., 2008; Sato et al., 2012) to spintronic-based magnetic racetrack memories (Arnold et al., 2006; Judy and Muller, 1996; Lenz, 1990), inductors (Popovic et al., 1996), actuators (Bozorth, 1951), sensors (Liu et al., 2004; Scheunert et al., 2016), and microelectromechanical systems (MEMS) and nanoelectromechanical systems (NEMS) (Cooper et al., 2005; Kim et al., 2014; Osaka, 2000; Romankiw, 1997). The most desirable soft magnetic properties are high saturation magnetization (M_s_), high permeability, low coercivity (H_c_), and low core loss. Thus, most soft magnetic materials are derived from the iron group metals (i.e., Ni, Fe, and Co).

Fe_X_Co_1-X_ binary alloys are important soft magnetic materials with excellent magnetic properties, including relatively low coercivity (∼2 Oe), low hysteresis loss, high electric permeability, high saturation magnetization (e.g., M_s_ of 2.4 T for Co_50_Fe_50_) (Shao et al., 2007; Shao et al., 2010), and relatively high electrical resistance (Andricacos and Robertson, 1998; Brankovic, 2012; Burkert et al., 2004; Cooper et al., 2005; Ehrfeld, 2003; Ghemes et al., 2017; Ghemes et al., 2017; Kohn et al., 2001; Sahari et al., 2006; Turgut et al., 1998; Zhan et al., 2002). Especially, nanocrystalline Fe_X_Co_1-X_ alloys are highly desirable materials for high-temperature applications, such as magnetic bearings for high-speed motor, flywheels (Yu et al., 2000), and gas turbine engines (Fingers et al., 1999; Giri et al., 2000; Kortus et al., 2002; Shang C. H. et al., 2000; Shang C.-H. et al., 2000; Turgut et al., 2000).

Although many synthesis methods are available for the preparation of Fe_X_Co_1-X_ films (Ashar, 1997; Shao et al., 2003), electrodeposition is an important processing technology because of its low capital/equipment and operating cost, high yield, low energy consumption, fast deposition rates, ability to handle complex geometries, high scalability, and capability (Dini, 1993; Wu et al., 2016; Wu et al., 2017). In addition, the material properties (e.g., morphology, composition, crystallinity, and crystal structures) can be readily tailored by controlling the electrodeposition parameters (Bai and Hu, 2003; Kockar et al., 2010; Lallemand et al., 2004).

Electrodeposition of Fe_X_Co_1-X_ has been investigated by many groups. Most of Fe_X_Co_1-X_ binary alloys were electrodeposited in acidic aqueous baths, with Fe^2+^ and Co^2+^ being used as precursors in aqueous media. In these works, solution parameters such as pH (Brankovic et al., 2006) and additives (Brankovic et al., 2008) and electrodeposition parameters such as deposition potential (Brankovic et al., 2009) were adjusted to control the film morphology and microstructures, which resulted in different magnetic properties. With respect to acidic baths, several major challenges need to be overcome to achieve high saturation magnetization (M_s_ ≥2.4 T). First, Fe^3+^ ions are always present in the aqueous solution because of the oxidation of Fe^2+^ either by the dissolved oxygen from the air or by the anode surface during the Fe_X_Co_1-X_ thin-film electrodeposition (Elhalawaty et al., 2011; Elhalawaty et al., 2012; Lide, 2005; Mehrizi et al., 2012; Zhang and Ivey, 2004). Moreover, electrodeposition of Fe_X_Co_1-X_ films in an aqueous solution is a process where the dramatic hydrogen gas evolution reaction occurs in parallel, leading to the increase in local pH at the electrode/solution interface and precipitation of insoluble metal hydroxides, especially Fe(OH)_3_ because of its low solubility (Mehrizi et al., 2012), in the Fe_X_Co_1-X_ films (Brankovic et al., 2008; Zhang and Ivey, 2004). Precipitation of non-magnetic metal hydroxide particles would decrease the saturation magnetization of the aqueous solution and shorten the bath life (Brankovic et al., 2008; Osaka et al., 2003). It was reported that a critical concentration of Fe^3+^ >1.2 mM in solution results in a dramatic decrease in the M_s_ value of the CoFe alloy by ∼44% (George et al., 2013; Tabakovic et al., 2006). In addition, the oxygen content in a CoFe film should remain lower than 1%–3% to reach a high saturation magnetization (Brankovic et al., 2008; Elhalawaty et al., 2011; Gao et al., 2006; Riemer et al., 2009). As a consequence, the incorporation of Fe(OH)_3_ in the deposit is the major obstacle in obtaining Fe_X_Co_1-X_ films and nanostructures with high saturation magnetization (Shao et al., 2007) and low coercivity (Yue et al., 2009). The accumulation of Fe^3+^ ions in solution is normally prevented by continuous chemical reduction (e.g., L-ascorbic acid) of Fe^3+^ ions to Fe^2+^ ions (Abd El-Halim and Fawzy, 1993; Almasi Kashi et al., 2010; Brener, 1994; Elbaile et al., 2012; Esmaeily et al., 2013; Huang et al., 2010; Ji et al., 2014; Kim et al., 2012; Ramazani et al., 2011; Schlesinger and Paunovic, 2011; Viqueira et al., 2015; Yang et al., 2010). The second challenge in the electrodeposition of Fe_X_Co_1-X_ films in an aqueous solution is that they undergo anomalous co-deposition, in which the less noble metal (i.e., Fe) deposits preferentially (Osaka et al., 1999a; Popov et al., 1993; Schlesinger and Paunovic, 2000). Additionally, additives were normally used in aqueous solution to improve the brightness and crystal structure, achieve smaller grain size, and reduce the residual stresses in the deposit (Brankovic et al., 2007; Osaka et al., 1999a; Osaka et al., 2003). However, additive molecules or molecular fragments can be found in the deposits as well (Brankovic et al., 2005; Brankovic et al., 2007; Edwards, 1962; Frankel et al., 1993; George et al., 2008). For example, the existence of sulfur in the magnetic deposit from saccharin as an additive occurs either via saccharin adsorption–electroreduction or via its physical incorporation during the deposit growth (Osaka et al., 1999b; Osaka et al., 2003). The significant presence of the interstitials, such as boron, sulfur, metal sulfides, or S-containing organic molecules, can cause a deterioration in the alloy’s magnetic performance and corrosion resistance (Osaka, 2000; Osaka et al., 1998; Smith et al., 2014).

Deep eutectic solvents (DESs), a class of ionic solutions closely related to ionic liquids but contain organic components (e.g., urea, amide, and acid), have emerged as new electrolytes for electrodeposition because of their relatively low vapor pressure; high tolerance of humidity; good thermo-stability; high solubility of metal precursors including metal salts, metal oxides, and metal hydroxides (Abbott et al., 2003; Binnemans et al., 2015; Brankovic et al., 2006; Brankovic et al., 2008); and greater deposition potential windows compared to aqueous electrolytes (Brankovic et al., 2009). Other advantages of DESs compared to aqueous bath have been highlighted by many authors (Bockris and Conway, 1975; Dulal et al., 2007).

Miller et al. (2017) investigated electrodeposition of iron thin films from choline chloride–ethylene glycol, with FeCl_3_ as the iron precursor. They observed that the iron complex is strongly dependent on the chloride-to-iron ratio. For example, when the ratio is greater than 4, [FeCl_4_]^−^ and [FeCl_4_]^−2^ are dominant iron complexes, whereas ethylene glycol forms a complex with iron when the ratio is less than 4 (Endres et al., 2017). Yanai et al. reported the galvanostatic deposition of Fe_X_Co_1-X_ alloys at a fixed current density of 67 mA/cm^2^ from choline chloride–ethylene glycol electrolytes with FeCl_2_ and CoCl_2_ as metal precursors at 100 °C (Yanai et al., 2015). They demonstrated the ability to for electrodeposition of smooth Fe_X_Co_1-X_ thin films at high current efficiency (>90%). The magnetic saturation of the deposits was in good agreement with the Slater–Pauling curve.

Fe_X_Co_1-X_ thin films have been systematically electrodeposited in the choline chloride–urea DES with FeCl_3_ and CoCl_2_ as precursors. Unlike other reported data, various electroanalytical methods including linear sweep voltammograms (LSVs) and chronoamperograms (CAs) were utilized to investigate the electrodeposition mechanism. Furthermore, Fe_X_Co_1-X_ films were synthesized using a potentiostatic method under varying potentials and temperatures, and their effects on the composition, morphology, crystal structures, and magnetic properties were systematically investigated.

## Experimental procedure

The DES was prepared by mixing the choline chloride and urea (1:2 ratio) at 80 °C until completely liquefied. Additionally, anhydrous cobalt chloride (CoCl_2_) and anhydrous iron chloride (FeCl_3_) were added and dissolved in the DES. The concentrations of FeCl_3_ and CoCl_2_ were fixed at 85 and 15 mM, respectively.

Electrodeposition experiments were performed in a conventional three-electrode cell using a platinum-coated silicon wafer as the working electrode. Platinum-coated titanium stripes and silver were used as the counter and reference electrodes, respectively. The total charge was fixed at 6 C. Linear sweep voltammetry (LSV) was conducted to investigate the electrodeposition mechanisms of Fe_X_Co_1-X_ with a fixed scan rate of 1 mV/s. The effect of the applied potential and temperature was investigated by varying the applied potential from −0.7 to −1.0 V and the temperature from 70 °C to 130 °C.

The morphology, composition, and crystal orientation of tellurium films were investigated by scanning electron microscopy (SEM, TESCAN VEGA), energy-dispersive spectroscopy (EDX, Ametek), and X-ray diffraction (XRD, PANalytical Empyrean) with 0.026° increments. The average grain size was determined using the Scherrer equation. The current efficiency (CE) was determined by measuring the mass of the electrodeposited Fe_X_Co_1-X_ films divided by the mass calculated from the charge based on CAs.

## Results and discussion

**FIGURE 1:**
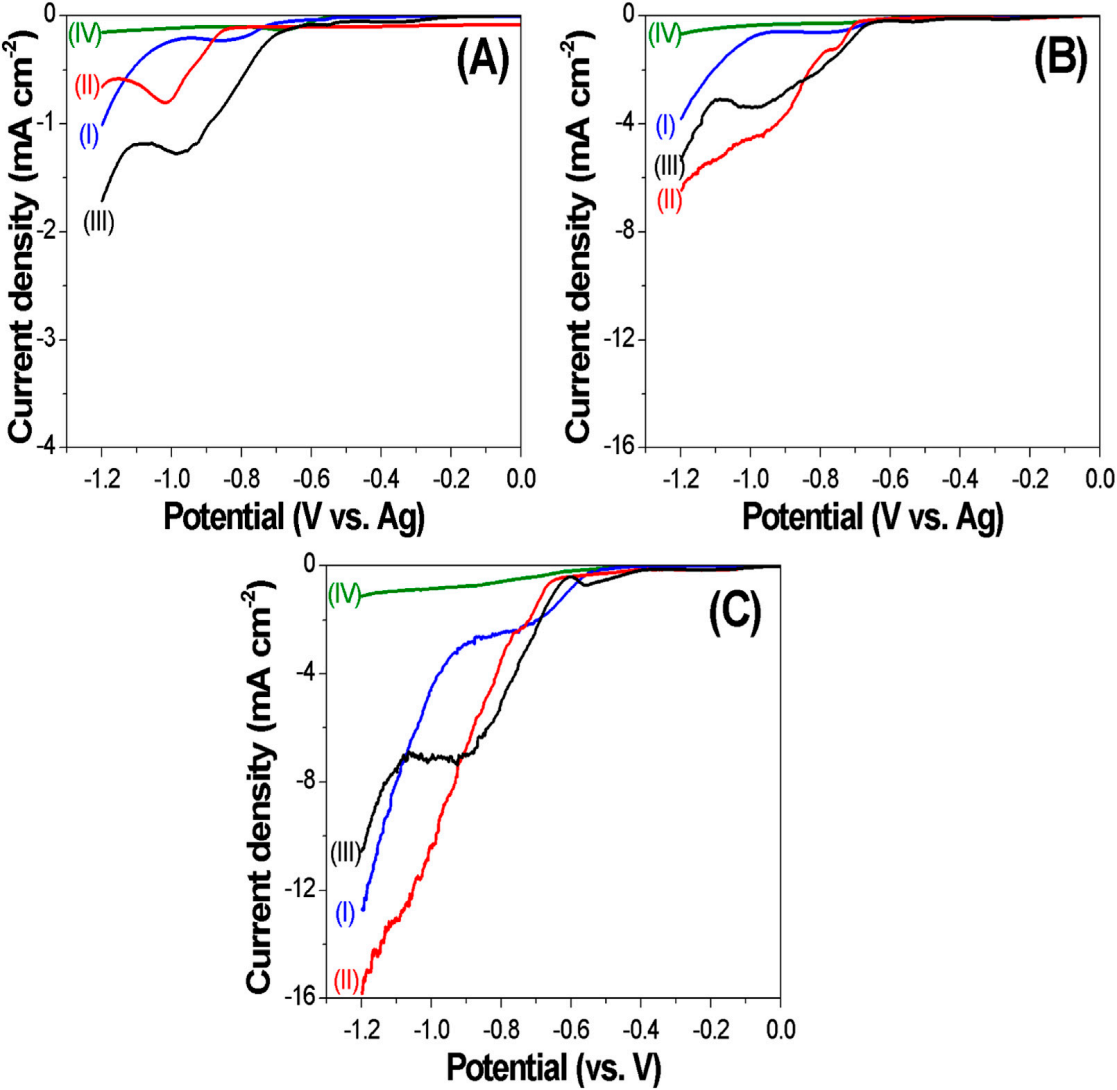
Linear sweep voltammograms for Co, Fe, and CoFe electrodepositions in choline chloride–urea at different temperatures: **(A)** 70 °C; **(B)**: 100 °C; **(C)**: 130 °C with (I) 15 mM CoCl_2_; (II) 85 mM FeCl_3_; (III) 15 mM CoCl_2_ + 85 mM FeCl_3_; (IV) only DES solvent. The scan rate was fixed at 1 mV/s using a silver wire as the reference electrode. As deposition temperature increased, the deposition rate of iron and cobalt significantly increased.

Figure 1 shows the temperature-dependent linear sweep voltammograms of electrolytes containing CoCl_2_ (I), FeCl_3_ (II), and CoCl_2_ and FeCl_3_ (III) and that without metal salts (IV) from 70 °C to 130 °C. In the absence of metal salts, the cathodic current density was relatively low, which indicates that there was a minor side reaction due to the decomposition of urea, where the onset potential of DES decomposition shifted positively from −0.56, −0.50, and −0.44 V as the temperature increased from 70 °C, 100 °C, and 130 °C, respectively. In the presence of metal salts, the current density significantly increased with increasing temperature at a fixed potential. For example, the electrolyte containing only CoCl_2_ as metal ions showed a reduction peak at the applied potential of approximately −0.8 V, which represents the electrochemical reduction of Co^2+^ to Co(s) (blue curve). The electrolyte only containing FeCl_3_ showed a cathodic peak at the applied potential of approximately −1.0V, which represents the electrochemical reduction of Fe^+3^ to Fe(s). As expected, the electrolyte containing both CoCl_2_ and FeCl_3_ shows two cathodic peaks. Additionally, at a fixed applied potential of −0.8 V, the reduction current density of Co increased from −0.2 to −0.61 to −2.5 mA cm^−2^, the current density of Fe increased from −0.1 to −1.4 to −3.4 mA cm^−2^, and the current density of Fe_X_Co_1-X_ increased from −0.55 to −1.9 to 5.0 mA cm^−2^ as the temperature increased from 70 °C to 100 °C to 130 °C.

Figure 1A shows that when the applied potential changed from −0.7 to −0.8 V, the average current density of Co increased from −0.07 to −0.20 mA cm^−2^, but when the applied potential further varied to −0.9 and −1.0 V, the current density of Co deposition remained at approximately 0.22 mA cm^−2^. As shown in the LSV curve I in Figure 1A, in the applied potential range of −0.8 to −1.0 V, the electrodeposition of Co reached a limiting current, where the electrochemical reduction reaction altered from kinetic control to mass transfer control. For iron electrodeposition at 70 °C, the current density continuously increases when the applied potential becomes more negative (Figure 1A; curve II). This is probably the reason why when the applied potential became more negative, the Fe content increased. In the acidic baths, anomalous co-deposition is observed, and Fe, the less noble metal, is deposited preferentially (Dulal et al., 2007). In DES, the same phenomenon was observed. For example, at an applied potential of −0.8 V at 70 °C, according to the LSV data (Figure 1A), the current density of Co and Fe deposition was −0.20 and −0.11 mA cm^−2^, respectively. However, the Fe content of the Fe_X_Co_1-X_ film deposited at −0.8 V and 70 °C is 56%, which means that the less noble metal (i.e., Fe) was preferentially electrodeposited.

**FIGURE 2:**
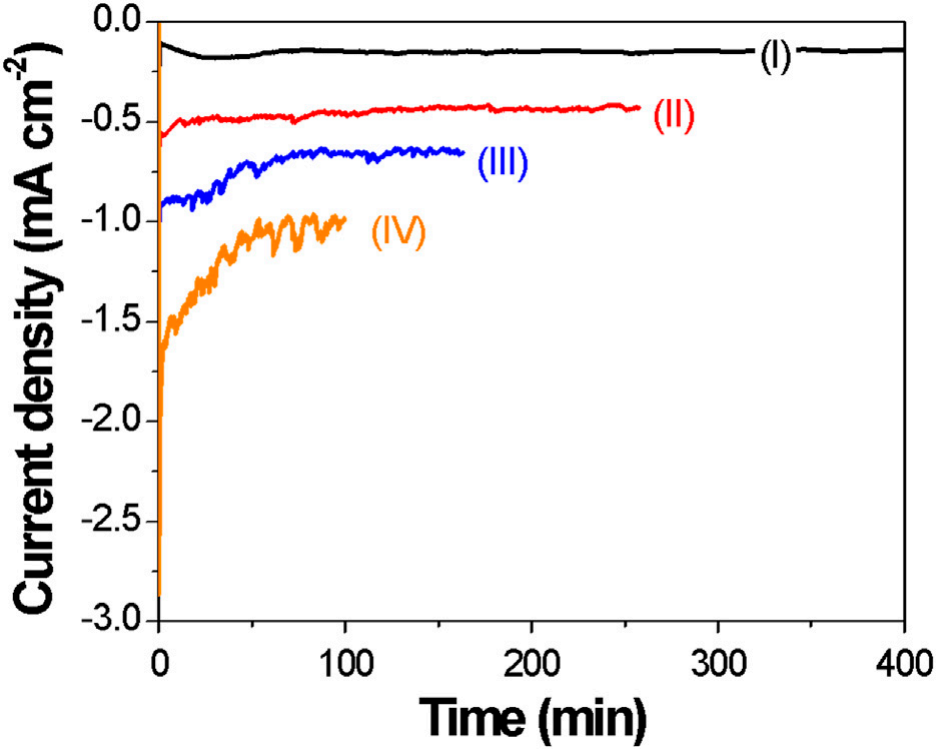
Chronoamperograms of Fe_x_Co_1-x_ electrodeposition at different applied potentials: (I) −0.7 V; (II) −0.8 V; (III) −0.9 V; (IV) −1.0 V. Temperature and the applied charge were fixed at 70 °C and 6 °C, respectively. A silver wire was used as the reference electrode. The deposition current increased with an increase in cathodic potential.

It is well-known that the magnetic properties of Fe_X_Co_1-X_ films are greatly affected by their compositions and microstructures (Dulal et al., 2007; Natter and Hempelmann, 1996; Wu et al., 2016); thus, a reliable control of the composition and microstructure is essential. The effect of the applied potential on electrodeposition of Fe_X_Co_1-X_ thin films was investigated under potentiostatic conditions at 70 °C. As shown in the CAs (Figure 2), current transients are relatively constant at the low applied potential of −0.7 and −0.8 V. However, at a higher applied potential (e.g., −0.9 and −1.0 V), the current transients started to fluctuate.

**FIGURE 3:**
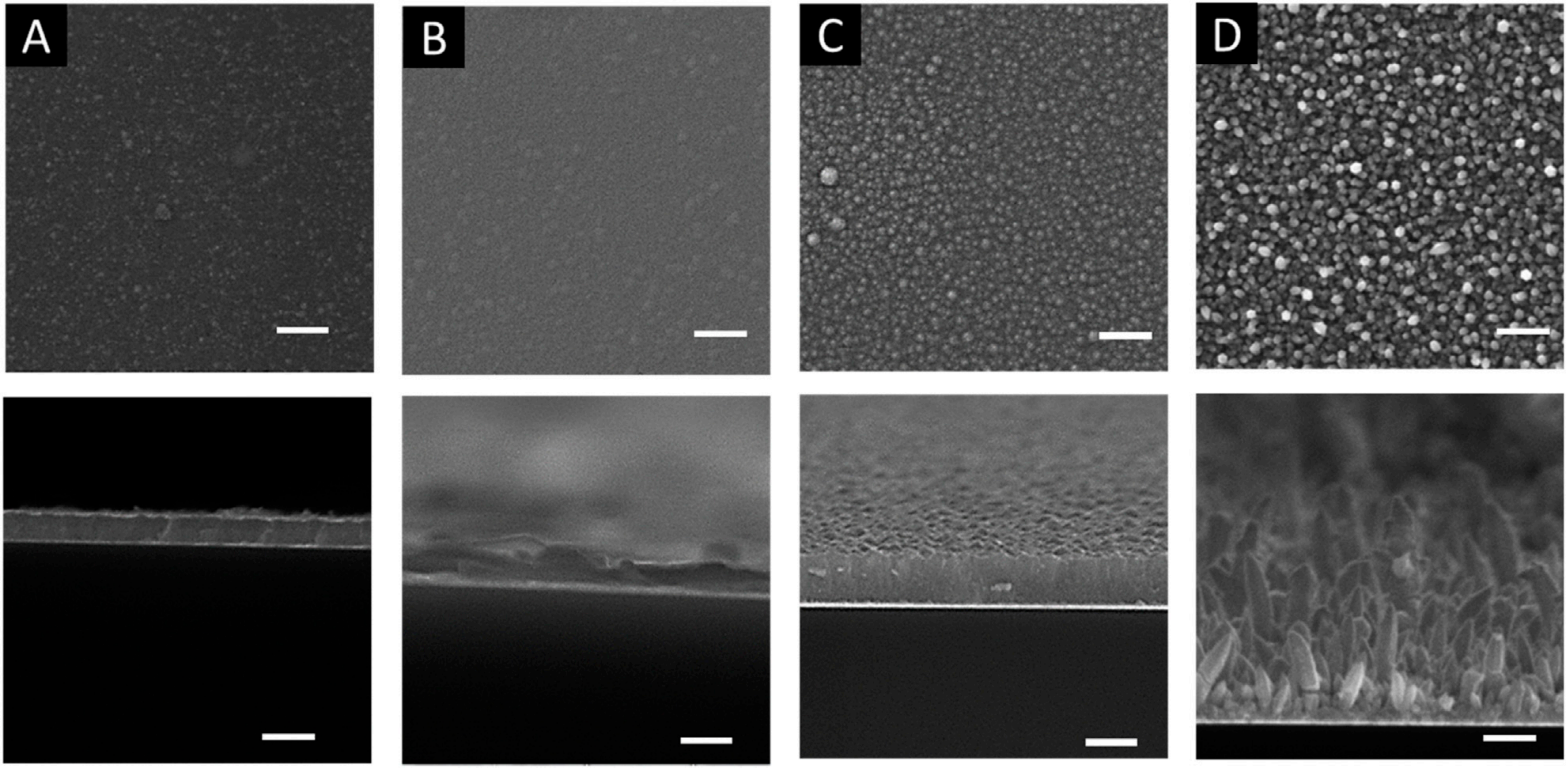
SEM images of Fe_x_Co_1-x_ electrodeposited at different applied potentials: **(A)** −0.7 V; **(B)** −0.8 V; **(C)** −0.9 V; **(D)** −1.0 V with 15 mM CoCl_2_ + 85 mM FeCl_3_ at the temperature of 70 °C. The top row images are the cross-sectional view, and the bottom row images are the top view. The length bars represent 2 microns. The morphology significantly changed with applied potential from smooth to nodular morphology.

Figure 3 shows the top (top row) and cross-sectional (bottom row) images of electrodeposition of Fe_X_Co_1-X_ thin films. At an applied potential of −0.7 to −0.9 V, the cross-sectional images (Figure 3 bottom row) showed that the electrodeposited films were compact with nodular surface morphology (Figure 3 top row). The nodular size increases with increasing applied potential. At an applied potential of −1.0 V, the morphology changed to a nanorod array with the average diameter of approximately 400 nm. According to the LSV curve III in Figure 1A, when the applied potential was more negative than −0.96 V, the deposition current reached a limiting current, indicating that mass transfer of metal ions limits the electrochemical reaction. Consequently, with the applied potential of −1.0 V, the morphology of electrodeposited Fe_X_Co_1-X_ was no longer a compact film. Fe_X_Co_1-X_ deposited in DES at −1.0 V showed a nanorod array instead of a porous or dendritic morphology, which are typically observed in electrodeposits under mass transfer limits (Dulal et al., 2007).

**FIGURE 4:**
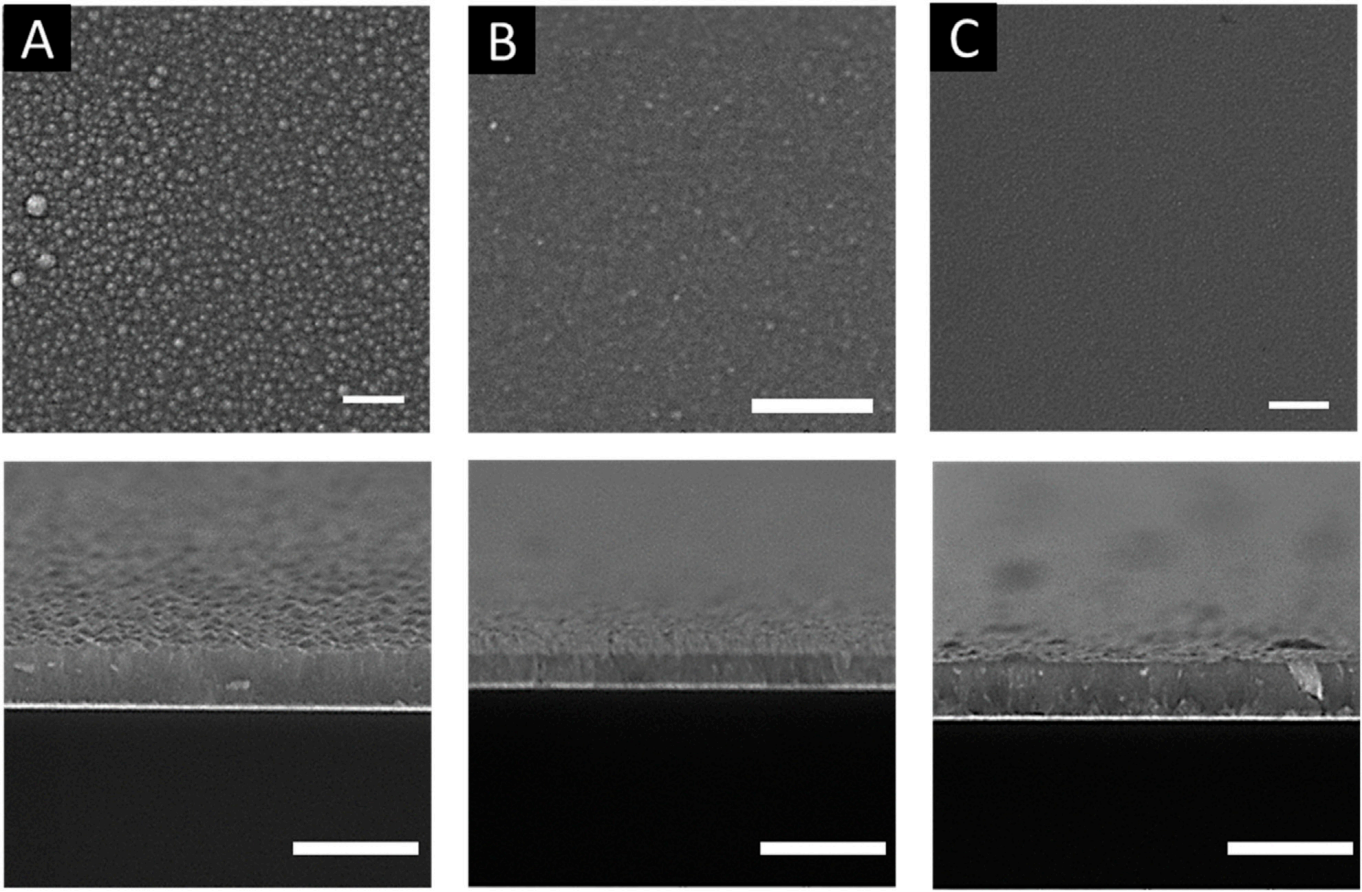
SEM images of Fe_x_Co_1-x_ electrodeposited at different temperatures: **(A)** 70 °C; **(B)** 100 °C; **(C)** 130 °C with 15 mM CoCl_2_ + 85 mM FeCl_3_ at the applied potential of −0.9 V. The top row images are the cross-sectional view, and the bottom row images are the top view. The length bars represent 2 microns. As the deposition temperature increased, the film’s morphology became more smoother.

The electrodeposition temperature was found to significantly affect the morphology and magnetic properties of electrodeposited films (Dulal et al., 2007; Natter and Hempelmann, 1996). The effect of temperature on the electrodeposition of Fe_X_Co_1-X_ was investigated at the applied potential of −0.9 V by varying the temperature from 70 °C to 130 °C. The CA curves at different temperatures (i.e., 70 °C, 100 °C, and 130 °C) are shown in Figure 3. According to the figure, the current density increased with increasing reaction temperature, and the CA curves fluctuated at higher temperatures. The effects of the reaction temperature on the morphology of Fe_X_Co_1-X_ films are presented by SEM images in Figure 4. As shown in the figure, the surface morphology of the films was smoother when the temperature was increased from 70 °C to 130 °C.

**FIGURE 5:**
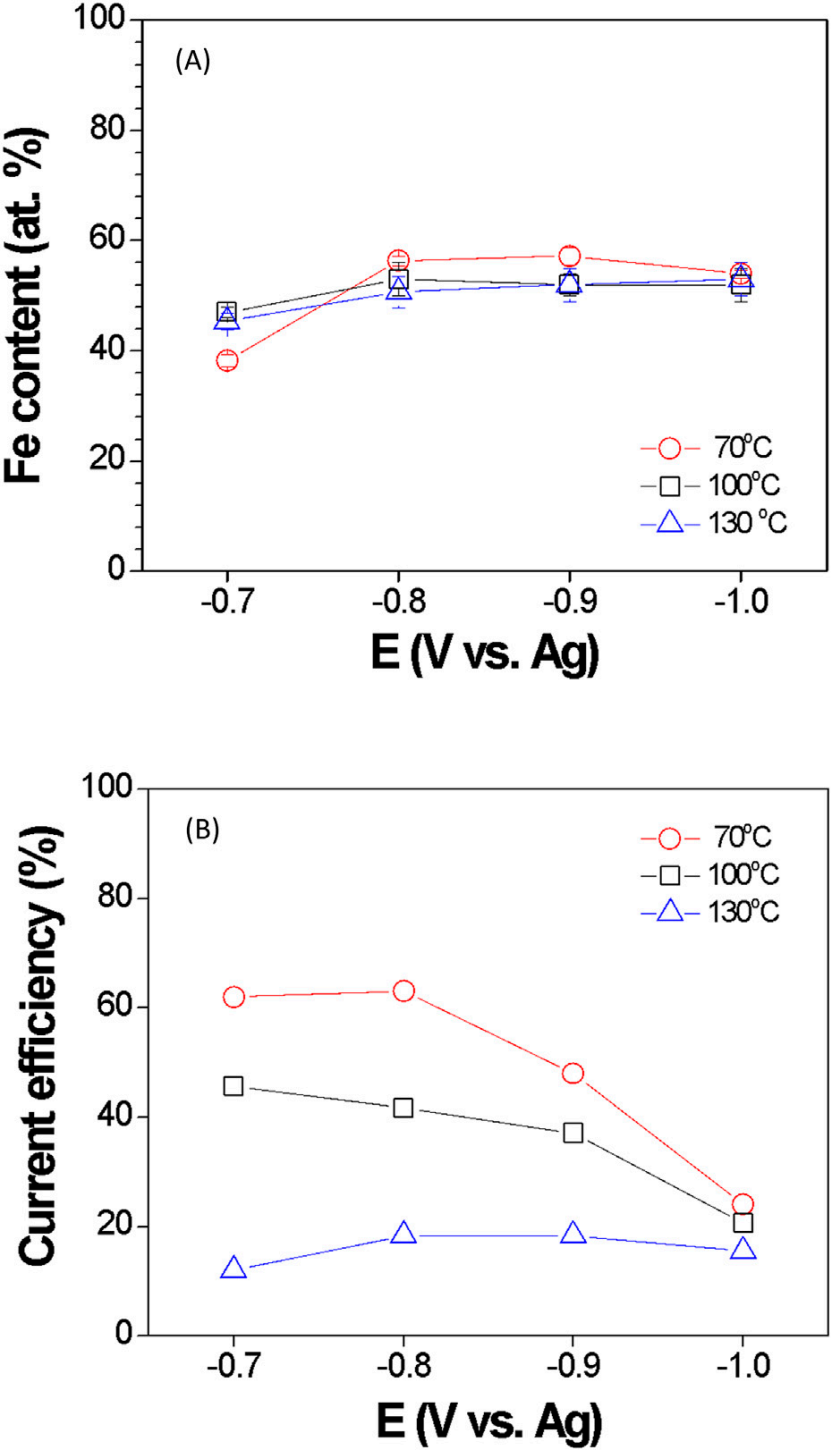
Deposited Fe content **(A)** and current efficiency (CE) **(B)** of Fe_x_Co_1-x_ thin films electrodeposited at different applied potentials. The electrolyte consisted of 15 mM CoCl_2_ + 85 mM FeCl_3_. The CE decreased with an increase in the deposition temperature, whereas the Fe content was independent of temperature.

**FIGURE 6:**
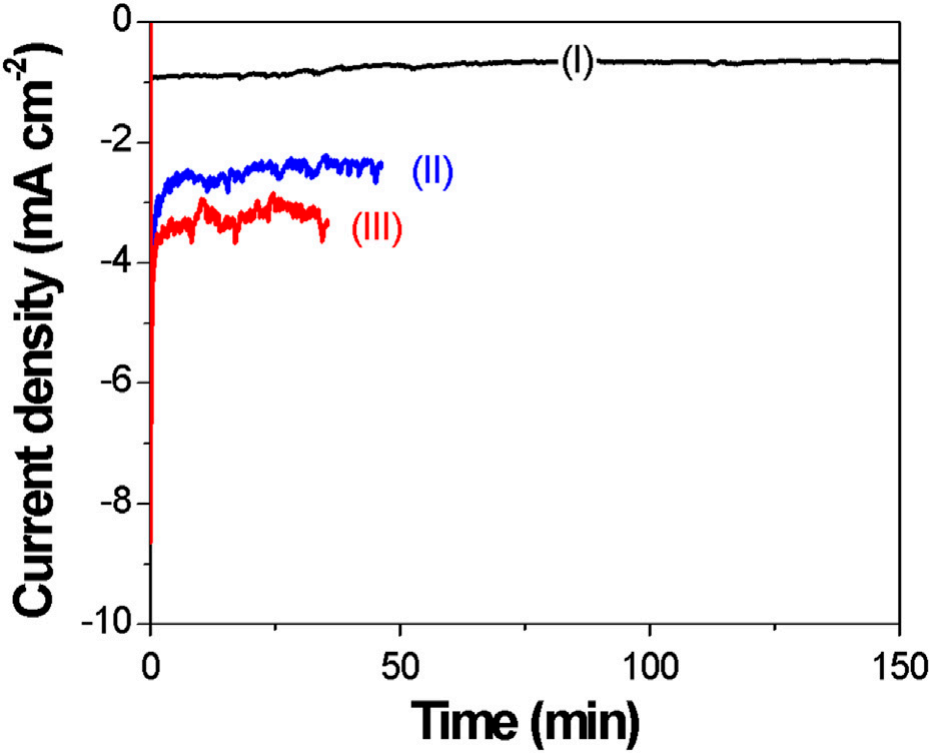
Chronoamperograms of Fe_x_Co_1-x_ electrodeposition at different temperatures: (I) 70 °C; (II) 100 °C; (III) 130 °C in the solution with 15 mM CoCl_2_ + 85 mM FeCl_3_. The applied potential and charge were fixed at −0.9 V and 6 C, respectively. A silver wire was used as the reference electrode. As the deposition temperature increased, the deposition current increased as well.

The effects of the applied potential and temperature on Fe content are shown in Figure 5. At 70 °C, the Fe content increased from 38 at. % to 56 at. %, when the applied potential was varied from −0.7 to −0.8 V; when the applied potential further increased to −0.9 V, the Fe content increased slightly to 57 at. %; however, at the applied potential of −1.0 V, the Fe content decreased to 54 at. %. At 100 ^o^C, the Fe content increased from 47 at. % to 53 at. % when the applied potential was changed from −0.7 to −0.8 V; however, the Fe content remained at 52 at. %, when the applied potential was changed from −0.9 to −1.0 V. At 130 °C, the Fe content increased significantly from 45 to 51 at. %, when the applied potential was changed from −0.7 to −0.8 V; however, when the applied potential varied from −0.9 to −1.0 V, the Fe content increased slightly from 52 at. % to 53 at. %, respectively. In general, the increase in Fe content as a function of overpotential is consistent with LSV data, in which, at higher overpotential, the current density of Fe deposition is higher, while the current density of Co electrodeposition remains approximately constant (Figure 1). Unlike the deposited Fe content, the current efficiency significantly depended on the operating temperature and applied potential, where it decreased with the increase in temperature and cathodic potential (Figure 6B).

It is a well-known phenomenon that the less noble metal (Fe) deposits preferentially over the more noble metal (Co) in an aqueous solution, primarily due to complexation and adsorption effects (Gonçalves et al., 2023; Zhou et al., 2012). Ferric ions can form complex ions with chloride, with the most common species in the aqueous solution being [FeCl_4_]^-^. At high chloride concentrations and under acidic conditions, the formation of [FeCl_4_]^-^ is favored. This can shift the redox equilibrium between ferrous and ferric ions to the left, as shown in Equation 1, effectively increasing the electrode potential of the Fe^3+^/Fe^2+^ couple. Additionally, the formation of such complexes further influences the redox potential.

$${\text{Fe}}^{3+}+e^{‐}\;⇌\;{\text{Fe}}^{2+}.$$ (1)

Similarly, cobalt(II) ions can also form complexes with chloride ions, particularly [CoCl_4_]^2-^. The formation of [CoCl_4_]^2-^ also contributes to a shift in the redox potential.

Anomalous electrodeposition in DESs has also been reported in the literature. The underlying causes are similar to those observed in aqueous solutions, where anomalous deposition is associated with ion complexation and reaction kinetics (Doneux et al., 2024). Additionally, mass transport limitations because of the high viscosity of DESs can restrict ion diffusion, thereby favoring the deposition of metal ions with faster electron transfer kinetics (Bernasconi et al., 2017).

**FIGURE 7:**
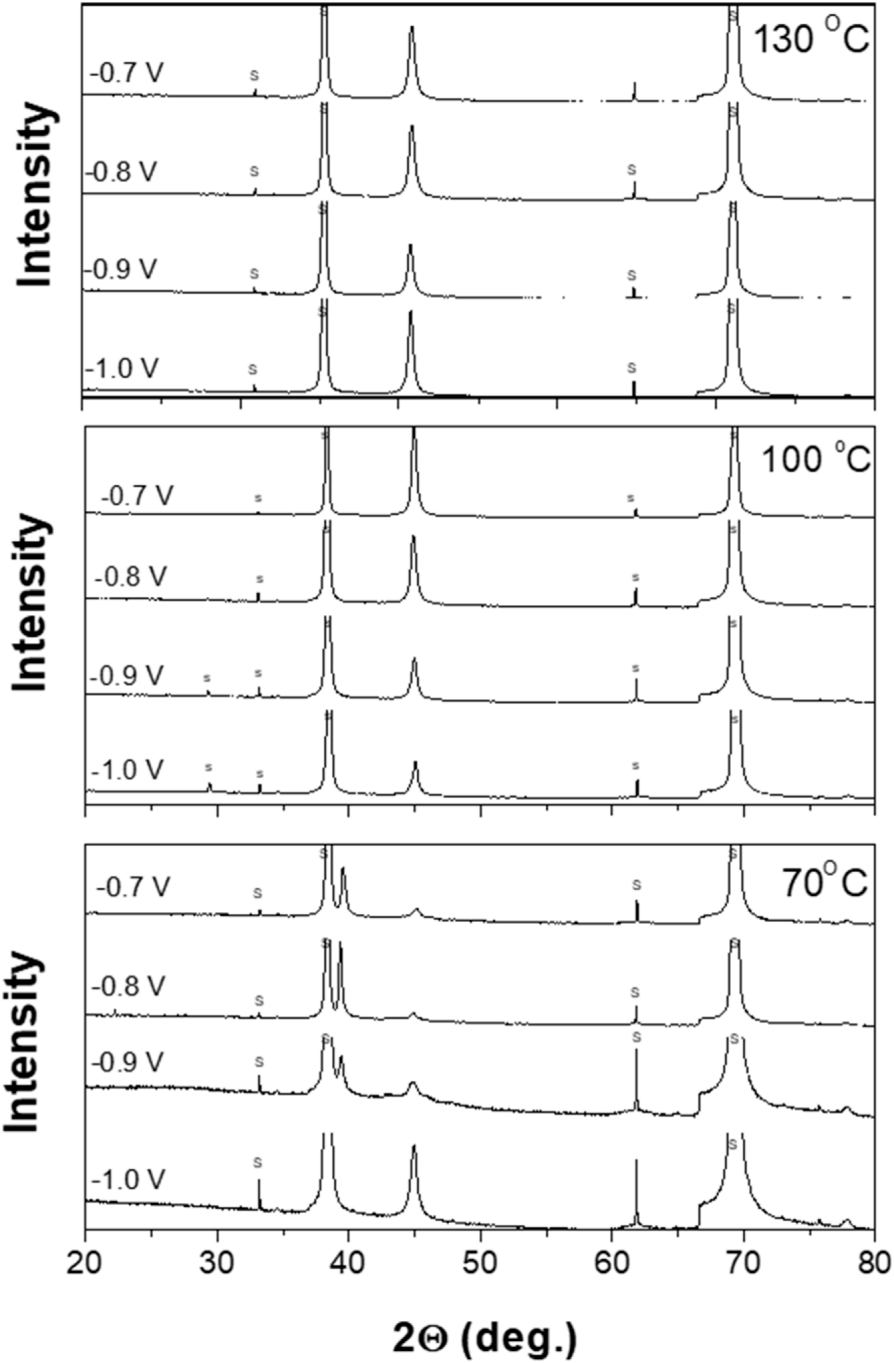
X-ray diffraction patterns of electrodeposited Fe_x_Co_1-x_ at different operating temperatures of applied potentials. At low temperature, iron oxides were co-deposited with the metallic film. At higher operating temperatures, metallic films were formed.

Figure 7 shows the XRD patterns of electrodeposited Fe_X_Co_1-X_ films as a function of the applied potential at a fixed temperature of 70 °C. Electrodeposited films from −0.7 to −0.9 V showed (110) a peak from body-centered cubic (BCC) FeCo and a (200) peak from α-Fe_2_O_3_. However, at an applied potential of −1.0 V, the deposit only showed the BCC (110) peak. During the electrodeposition process, the Fe^3+^ ions will be absorbed to the electrode first, followed by electrochemical reduction at the electrode surface (Dulal et al., 2007). At a low applied potential, the electrochemical reduction rate might not be sufficient enough to reduce all the absorbed Fe^3+^ ions; therefore, a certain amount of Fe^3+^ ions remained in the electrodeposited Fe_X_Co_1-X_, which was confirmed by the α-Fe_2_O_3_ peak. At an applied potential of −1.0 V, the reaction became mass transfer control, and the electrochemical reaction was high enough to reduce all of the absorbed Fe^3+^ ions on the electrode. This is probably the reason why there is no Fe_2_O_3_ peak in the XRD data at an applied potential of −1.0 V.

As the deposition temperatures increased to 100 °C and 130 °C, the XRD patterns only showed a (110) peak. This is probably because the high temperature results in a higher electrochemical reaction rate, which makes it fast enough to reduce all the Fe^3+^ ions absorbed on the electrode.

The average grain size of the electrodeposited Fe_X_Co_1-X_ films was estimated by the Scherrer equation. At the temperature of 70 °C, the average grain size of Fe_X_Co_1-X_ was ∼35 nm at the applied potentials of −0.7 and −0.8 V; when the applied potential became more negative to −0.9 and −1.0 V, the average grain size reduced to ∼30 nm. At 100 °C, the average grain size increased from 31 to 37 nm when the applied potential was increased from −0.7 to −0.8 V; however, when the applied potential further increased from −0.9 to −1.0 V, the average grain size maintained at approximately 37 nm. At the temperature of 130 °C, the average grain size of Fe_X_Co_1-X_ was approximately 35 nm when the applied potential was −0.7 and −0.8 V, and it increased to 39 nm when the applied potential was changed to −1.0 V. In summary, the overall variation of Fe_X_Co_1-X_ grain size as a function of the applied potential and temperature is small, which is from ∼29 to 39 nm.

**FIGURE 8:**
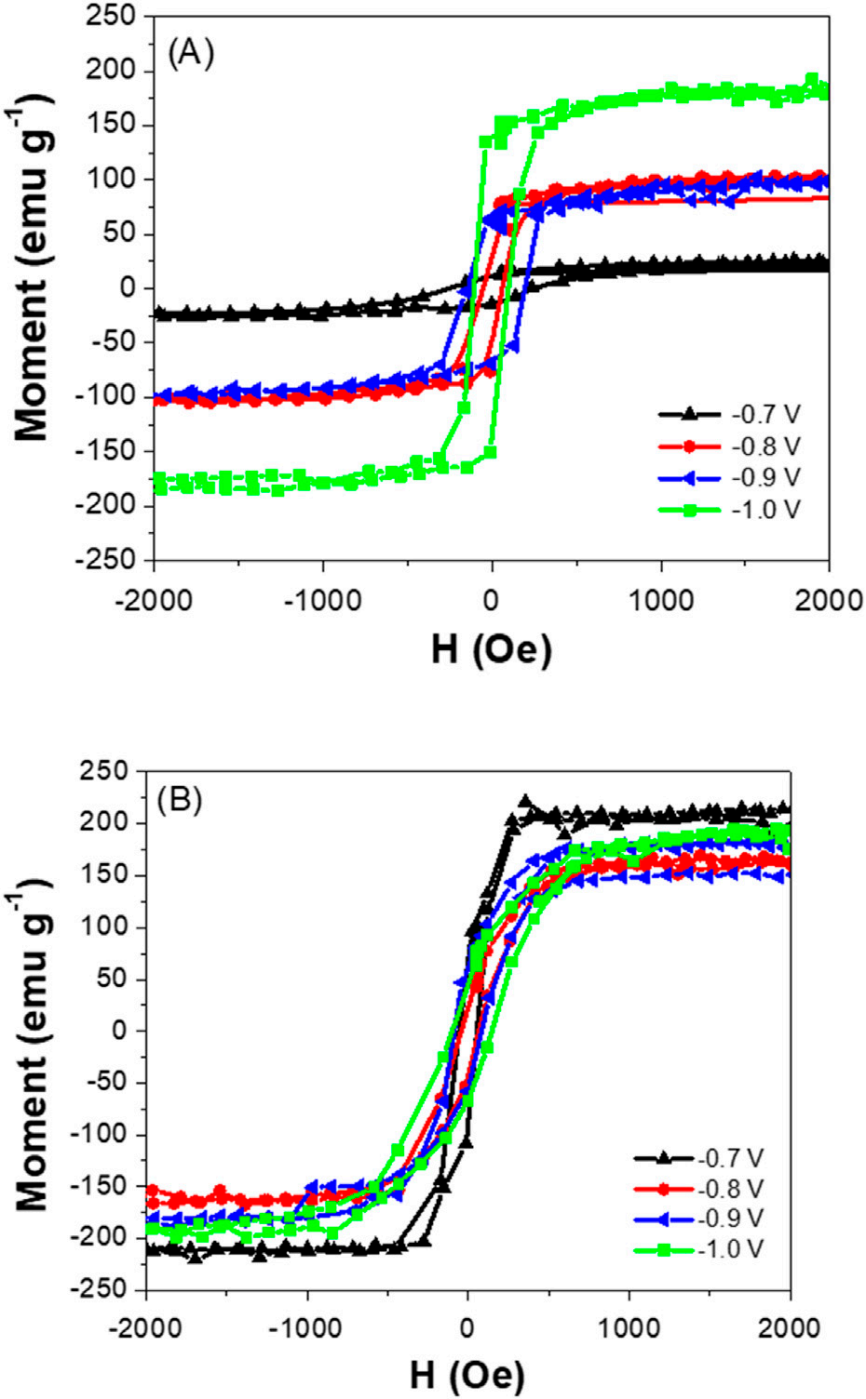
Parallel magnetic hysteresis loops of Fe_x_Co_1-x_ thin films electrodeposited at different temperatures (i.e., 70 °C **(A)** and 130 °C **(B)**). Magnetic properties are strongly influenced by impurity, composition and morphology.

**FIGURE 9:**
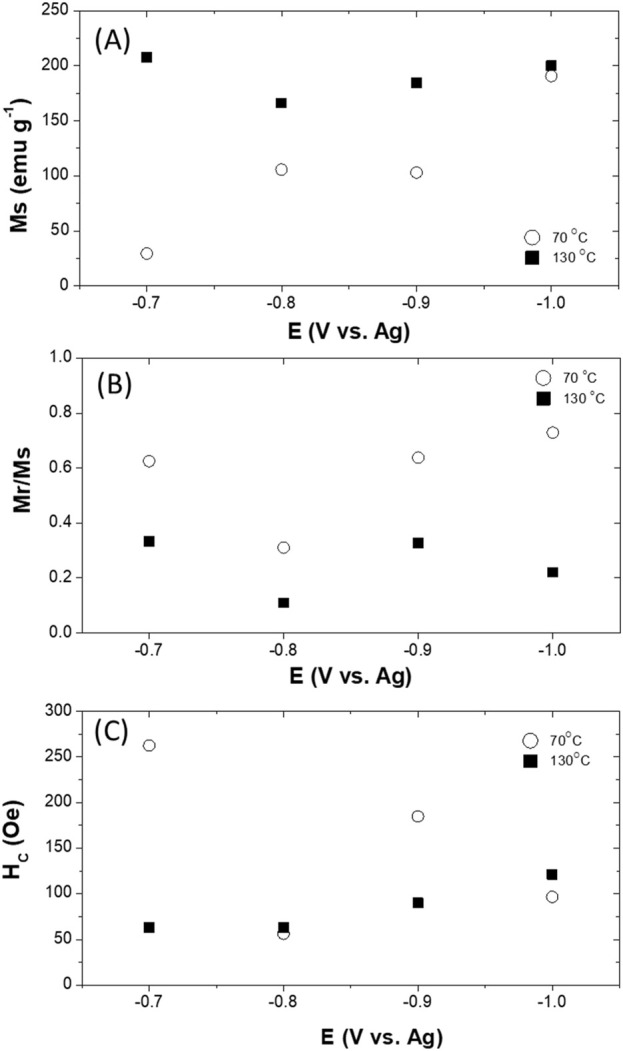
Magnetic saturation **(A)**, remanence **(B)**, and coercivity **(C)** of electrodeposited Fe_x_Co_1-x_ at different operating temperatures of applied potentials.

Figure 8 shows the parallel magnetic hysteresis loops of Fe_x_Co_1-x_ thin films electrodeposited at different temperatures (i.e., 70 °C and 130 °C). As expected, magnetic saturation (M_s_) showed a monotonic increase with increasing cathodic potential at 70 °C, whereas M_s_ was less dependent on the applied potentials (Figure 9A). The lower M_s_ may be attributed to the presence of α-Fe_2_O_3_ in the deposit. At high deposition temperature, only metallic Fe_x_Co_1-X_ were electrodeposited with similar composition, resulting in similar M_s._ As shown in Figures 9B, C, composite electrodeposited films showed greater squareness (M_r_/M_s_) and higher coercivity due to the co-existence of α-Fe_2_O_3_ in the deposit.

Supplementary Table S1 compares the magnetic properties of electrodeposited FeCo thin films from both aqueous and DES baths. As shown in the table, the intrinsic magnetic saturation (M_s_) strongly depends on the film composition, regardless of the bath type. However, extrinsic coercivity (H_c_) is highly influenced by both the composition and deposition conditions, including the nature of the electrolyte solution (i.e., aqueous or DES).

## Conclusion

Fe_X_Co_1-X_ thin films were electrodeposited in a DES solution using Fe^3+^ and Co^2+^ as precursors without reducing agents or other additives. The films had a smooth compact morphology when the applied potential was at the kinetic controlled range, while the morphology shifted to a nanorod array when the deposition was carried out under mass transfer control. The composition of Fe_X_Co_1-X_ films and current efficiency can be altered by varying the applied potential and deposition temperature. Generally, the Fe content increases first and then reaches a plateau when the applied potential varies from −0.7 to −1.0 V. The increase in Fe content as a function of overpotential may be caused by a higher Fe deposition rate at high overpotential, which is consistent with LSV data. Most of the Fe_X_Co_1-X_ electrodeposits from the DES show a body-centered cubic crystal structure with the preferred orientation of (110). All electrodeposited Fe_X_Co_1-X_ films had small grains ranging from 29 to 39 nm. At low temperatures and low cathodic potentials, co-deposition of iron oxide was observed. At higher cathodic potentials and elevated operating temperatures, the co-deposition of iron oxide was minimized or completely suppressed. The magnetic properties were strongly influenced by the presence of iron oxide.

## Data Availability

The original contributions presented in the study are included in the article/Supplementary Material, further inquiries can be directed to the corresponding authors.
