# Supplementary material for: Electrodeposition of nanocrystalline FeXCo1-X thin films from choline chloride–urea deep eutectic solvents
Source: Front Chem. 2025 Sep 1;13:1635084. doi: 10.3389/fchem.2025.1635084 (PMC12460894; doi:10.3389/fchem.2025.1635084)
Supplement: Supplementary file 1 [file DataSheet1.pdf]

Supplementary Information

Electrodeposition of Nanocrystalline Fe_xCo_{1-x} Thin Films from Choline Chloride-Urea Deep Eutectic Solvents

Tingjun Wu[1,*], Jiwon Kim[2], Yong-Ho Choa[3], and Nosang V. Myung[4,5]

[1]Xiamen Institute of Rare earth Materials, No.258 Duishan Road, Jimei District 361021, Xiamen, Fujian, P. R. China

[2]Institute for Advanced Engineering, 175-28, Goan-ro, 51 Beon-gil, Baegam-myeon, Cheoin-gu, Yongin, Gyeonggi, Republic of Korea

[3]Department of Materials Science and Chemical Engineering, Hanyang University, Ansan 426-791, Korea

[4]Department of Chemical and Biomolecular Engineering,
[5] Department of Chemistry and Biochemistry
University of Notre Dame, Notre Dame, IN 46654

*Corresponding author: Tingjun Wu: E-mail: tingjun.wu@hotmail.com Tel: +86 13859929536

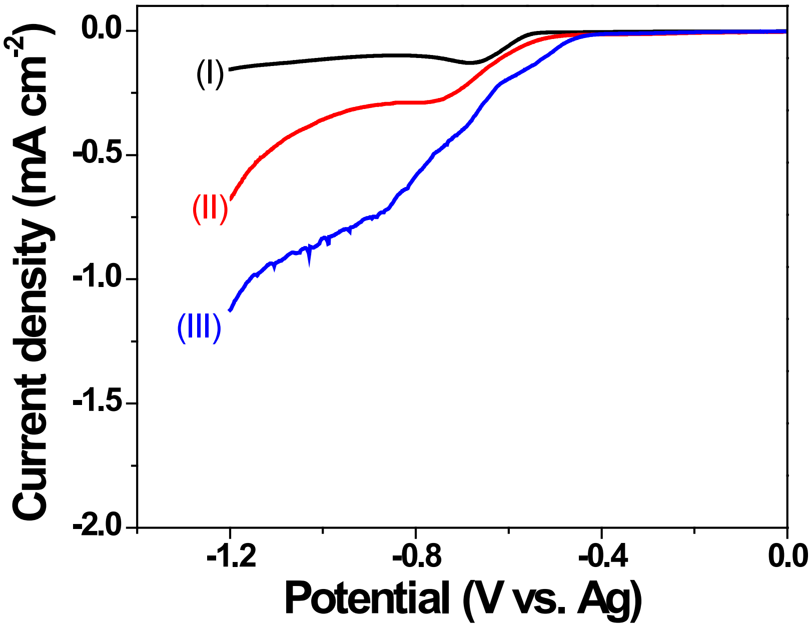

Figure S1. LSV curves with DES only at different temperatures (I): 70, (II): 100, (III): 130 °C. The scan rate was fixed at 1 mV/s.

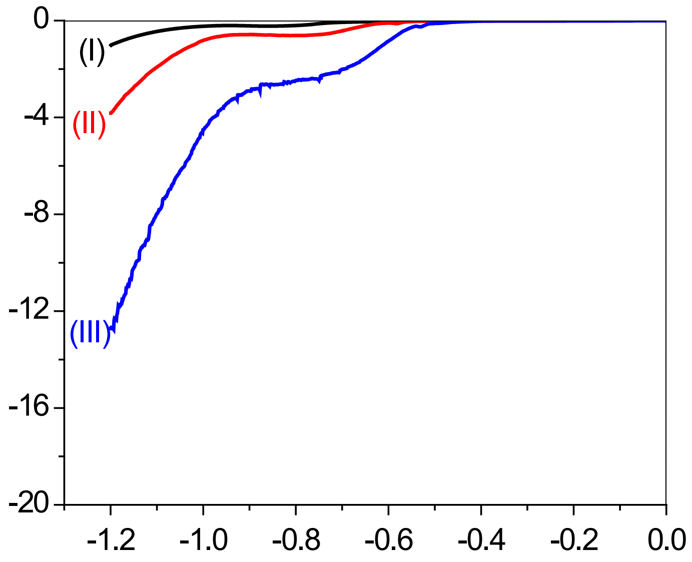

Figure S2. LSV curves for Co electrodepositions in DES at different temperatures (I): 70, (II): 100, (III): 130 °C, with 15 mM $CoCl_2$. The scan rate was fixed at 1 mV/s.

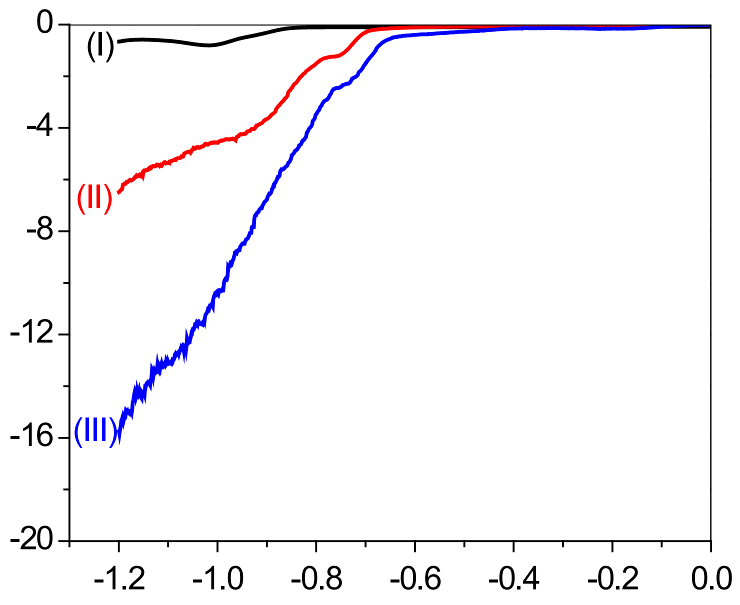

Figure S3. LSV curves for Fe electrodepositions in DES at different temperatures (I): 70, (II): 100, (III): 130 ºC, with 85 mM $FeCl_3$. The scan rate was fixed at 1 mV/s.

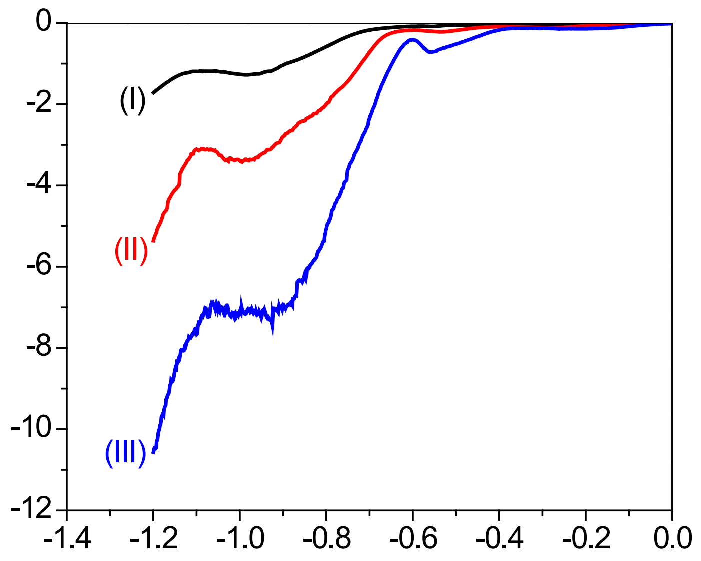

Figure S4. LSV curves CoFe electrodepositions in DES at different temperatures (I): 70, (II): 100, (III): 130 ºC, with 15 mM $CoCl_2$ + 85 mM $FeCl_3$. The scan rate was fixed at 1 mV/s.

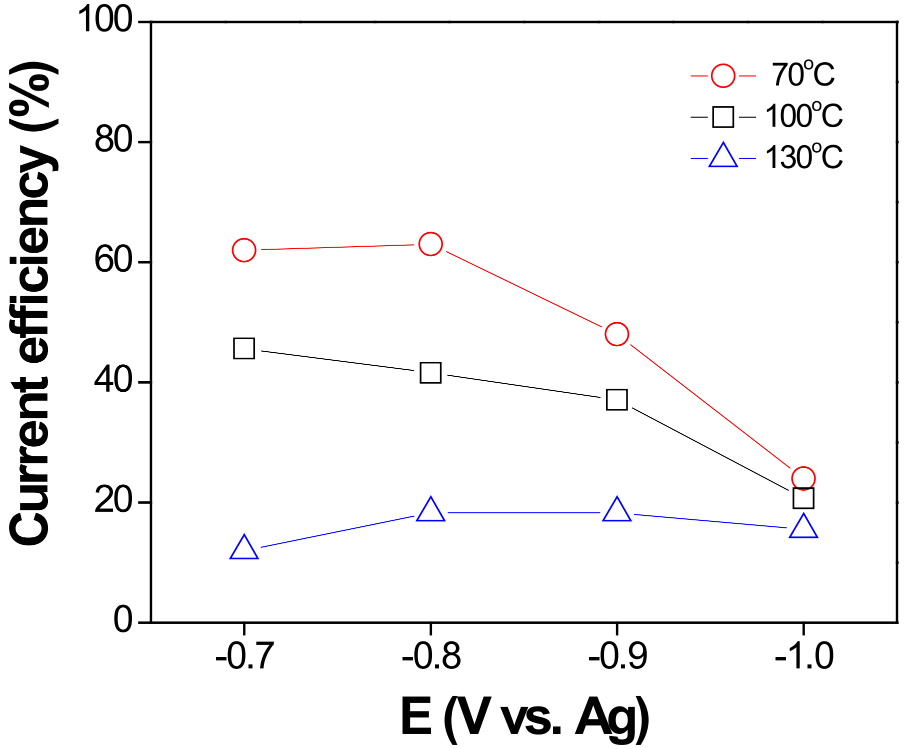

Figure S5. Current efficiency of CoFe film electrodeposited at different applied potential. The electrolyte consisted of 15 mM $CoCl_2$ + 85 mM $FeCl_3$.

Table S.1. Comparison of the magnetic properties of electrodeposited FeCo thin films from both aqueous and deep eutectic solvents baths.

Bath Type	***Fe content (at%)***	***Saturation Magnetization (M_s) (T)***	***Coercivity (Hc) (Oe)***	***Reference***
Aqueous	*60 - 80*	*1.8 – 2.4*	*50 – 250*	*Zhou et al., 2012*
Aqueous (Nanowires)	*70*	*~2.3*	*<100*	*DTIC Report, 2025*
Aqueous	*10 - 90*	*1.5 – 2.3*	*60 – 300*	*Gonçalves et al., 2023*
Aqueous	*65*	*2.1*	*80*	*Rahman et al., 2007*
Aqueous	*70*	*2.2*	*100*	*Ricq et al., 2001*
Aqueous	*0 - 10*	*1.8 to 2.4*	*8 to 12*	*Liao, 1987*
Aqueous	*0 - 80*	*2.3 to 1.5*	*20 - 62*	*Myung et al, 2004*
Aqueous	*55*	*2.2*	*30 – 60*	*Osaka et al., 1998*
Aqueous	*70*	*2.0*	*60 – 90*	*Lallemand et al., 2005*
Non-Aqueous (DES)	*75*	*1.9*	*50 – 100*	*Doneux et al., 2024*
Non-Aqueous (DES)	*65-85*	*0.8-1.74*	*290-350*	*Side et al., 2019*
Non-Aqueous (DES)	*30-40*	*0.2-1.6*	*50 – 250*	*This work*

References:

Doneux, T., Sorgho, A., Soma, F., Rayée, Q., & Bougouma, M. (2024). Electrodeposition in Deep Eutectic Solvents: The "Obvious", the "Unexpected" and the "Wonders". Molecules, 29(14), 3439.

DTIC Report. (2025). Electrodeposition of FeCo nanowires and their magnetic properties.

Gonçalves, S., Andrade, V., Sousa, C. T., et al. (2023). Tunable Iron–Cobalt Thin Films Grown by Electrodeposition. Magnetochemistry, 9(7), 161.

Lallemand, F., Ricq, L., Deschaseaux, E., et al. (2005). Electrodeposition and magnetic properties of FeCo alloys. Surface and Coatings Technology, 197(1), 10–16.

Liao, S. (1987). High moment CoFe thin films by electrodeposition. IEEE Transactions on Magnetics, 23(5), 2981–2983. https://doi.org/10.1109/TMAG.1987.1065555

Myung, N. V., Park, D.-Y., Urgiles, D. E., & George, T. (2004). Electroformed iron and FeCo alloy. Electrochimica Acta, 49(25), 4397–4404.

Osaka, T., Takai, M., Hayashi, K., et al. (1998). Electrodeposition of high-density magnetic recording media. Nature, 392(6679), 796–798.

Rahman, I. Z., et al. (2007). Magnetic properties of FeCo alloys. Journal of Physics: Conference Series, 61, 523.

Ricq, L., Lallemand, F., Gigandet, M. P., & Pagetti, J. (2001). Electrodeposition of FeCo alloys and their magnetic behavior. Surface and Coatings Technology, 138(2), 278–284.

Sides, W., Kassouf, N., & Huang, Q. (2019). Electrodeposition of FeCo alloys in ionic liquids. Journal of The Electrochemical Society, 166, D77.

Zhou, D., Zhou, M., Zhu, M., et al. (2012). Electrodeposition and magnetic properties of FeCo alloy films. Journal of Applied Physics, 111(7), 07A319.
